# Impact of a Web Program to Support the Mental Wellbeing of High School Students: A Quasi Experimental Feasibility Study

**DOI:** 10.3390/ijerph16142473

**Published:** 2019-07-11

**Authors:** Minna Anttila, Ruthaychonnee Sittichai, Jouko Katajisto, Maritta Välimäki

**Affiliations:** 1 Department of Nursing Science, University of Turku, 20014 Turku, Finland; 2 Kids and Youth Development Research Unit, Research Center for Educational Innovations and Teaching and Learning Excellence, Faculty of Humanities and Social Sciences, Prince of Songkla University, Muang, Pattani 94000, Thailand; 3 Department of Mathematics and Statistics, University of Turku, 20014 Turku, Finland; 4 School of Nursing, Hong Kong Polytechnic University, Hung Hom, Kowloon, Hong Kong, China

**Keywords:** information technology, adolescent, mental health, impact, acceptance, usability

## Abstract

Little effort has been made to investigate the potential of web programs aimed to support the mental wellbeing of adolescents in school environments in middle-income countries. A quasi-experimental feasibility study was conducted in Thailand with adolescents (N = 180) in three conveniently sampled high schools and with teachers (N = 12) who acted as program tutors. The web program was used in small groups, independently, or it was not used at all. No statistically significant changes were found between the groups regarding depression, stress, or satisfaction. Differences between program users (n = 61) and non-users (n = 48) were not significant. Acceptance was higher among adolescents who used the program independently (n = 40, 73% vs. n = 21, 39%; *p* = 0.001). Usability feedback did not differ between the groups. Support should be provided in order for programs to be potentially used. More information is needed regarding factors associated with the use of web programs.

## 1. Introduction

Modern technology has become crucial to adolescents for communicating and seeking information, including information about health and wellbeing. Adolescents especially use the web when seeking information about sex, human immunodeficiency virus (HIV), substance abuse [1], and mental health problems [2]. The web is a usable channel where one can avoid stigma, especially related to mental health problems [3,4]. Therefore, web use has become popular among adolescents with depression [3], and those who feel more comfortable communicating in writing [5]. 

A number of studies have tested the effectiveness of the computerized programs or web programs in preventing or reducing depressive symptoms in adolescents and young adults [6,7,8,9]. It has been found that web programs can be as effective as traditional face-to-face therapy [10]. Calear and Christenssen [11] conducted a systematic review in school-based prevention and early intervention programs to reduce depressive symptoms. Programs targeting students exhibiting elevated levels of depression were found to be the most effective (effect size range 0.21–1.40). Most programs were delivered by a mental health professional (or graduate student), and teacher-led programs were associated with fewer significant effects.

One of the concerns with using web programs in mental health intervention is delivery of the program, and participants’ low engagement and fidelity [12]. Cognitive-behavioral prevention programs conducted in school environments have particularly demonstrated low adoption and sustainability [13]. For example, in a study run in a school environment, Calear et al. [14] found that the completion rate of the six-week “Anxiety and Worry” program was higher if adolescents were supported by a health service provider. In Calear’s study, without the support of a health service provider, 78% (n = 333) of students only completed the first two weeks of the program at school, whereas 36% (n = 154) used all six of the designated weeks. However, when a health service provider lent support, 489 (87%) completed the first two weeks and 281 (50%) completed all six weeks [14]. Contradictory findings have also been reported. A study of “Stressbusters”, a program aimed at students with mild to moderate depression, found that 93% (n = 51) of the students completed at least half of the sessions and 86% (n = 47) completed all eight sessions in an assigned room at school. The program was delivered by headphone and CD-ROM (Compact Disc Read-Only Memory) on a laptop [15].

Indeed, different factors have been found to be associated with the participant adherence to web programs. Females and those who live in rural areas have been found to have greater adherence to programs run in school settings compared to those who access the program spontaneously or directly through an open-access Uniform Resource Locator (URL) [16]. In a study by Ho et al. [17], nine out of 13 participants used peer-networked online sessions within the first three weeks of a 10-week program. Qualitative interviews revealed that, at the beginning of the program, participants were willing to use peer networking features when site use by group members was high. However, a decrease in site use by group members during the subsequent weeks negatively affected their desire to log on or engage with the others [17]. The use of a technological application may decrease due to a lack of time, forgetfulness or doubt about the usefulness of the program [18]. Other reasons for non-use are technical problems, non-relevant topics or insufficient website content to sustain the participants’ interest [19].

Although in western society a wide range of studies about adolescents’ web use to support their wellbeing have already been conducted [20], coherent information is still needed on how web programs can be used to support adolescent wellbeing in developing countries [21]. More investment in mental health is needed, for example in Thailand, as the country has a young population; 18% are under 15, compared to 15% being over 60 [22]. Even though Thailand has achieved impressive achievements in economic and social development [23], a number of health concerns involving adolescents exist, such as being overweight among boys [24], pregnancy for 13 to 18-year-old girls [25], sexually transmitted infections [26,27], and mental health concerns [28]. More risk factors have been identified such as having poor relationships and suffering from loneliness and poor self-esteem that make it more likely for an adolescent to exhibit untoward behavior, poor school performance or early school leaving [1]. In Thailand, the overall prevalence rate for mental health problems is estimated to be 37.6% [29], while the prevalence of depressive symptoms is around 20%, although it varies between regions [28]. They have fewer mental health resources (psychiatrist and nurses) than the global average per 100,000 people [30].

A range of concerns has also been raised about how to better understand technology that mediates the provision of youth services [31]. We therefore aim to increase the knowledge about the potential of web programs in areas where technological solutions have rarely been used to support the wellbeing of adolescents. In this quasi-experimental study in Thailand, we (1) explore the impact of a web program aimed to support adolescent mental wellbeing at school. We also (2) compare program users and non-users, (3) identify engagement levels of adolescents using the program, and (4) consider adolescents’ and teachers’ feedback of the program. 

## 2. Materials and Methods

### 2.1. Design

The multicentre parallel quasi-experimental cluster design study with a pre-post design was run to assess the impact of a web program aiming to support adolescents’ mental wellbeing in a school environment. We assumed that if adolescents were to benefit especially from the peer-network possibility in small groups (active control) or either independent web program use over a six-week period, positive results would be seen in the form of decreased depression and stress scores and increased satisfaction levels regarding the services. Beyond the quasi-experimental study, a comparison between program users and non-users was carried out, and an evaluation of the acceptance and usability of the program was assessed from the participants’ point of view using multiple methods.

### 2.2. Participants

#### 2.2.1. Eligibility

The schools were purposively convenience sampled based on the previous knowledge of the researcher on our team from Thailand, and as the schools and study systems vary greatly depending on the geographic area. The selected schools for the study included both public and private schools with similar curricula and similar numbers of students, and were located in the same city area. Private primary and secondary schools located in the countryside were excluded. Participating adolescents were 15 to 19 years old, could read, write and speak Thai, and were able to voluntarily provide written informed consent for participation. Adolescents who had inadequate Thai skills or were unable to provide informed consent were excluded. Volunteer teachers were invited to participate in the study as tutors for the web program.

#### 2.2.2. Settings and Locations

The trial was undertaken at three high schools in Southern Thailand. The schools were located in the same city, which had around 160,000 inhabitants [32]. In total, there were 45 high schools in the province that offered education to 18,571 children. Out of them, 4894 adolescents were aged 15 to 18 years. In Thailand, education is provided mainly by the Thai government [32]. Public schools are part of basic education and are administered by the government. The national constitution guarantees children a free basic education at school (books, milk, not meals). A minimum of nine years of school attendance is compulsory. Education consists of at least twelve years of free education, including basic and higher education, and includes three years of both lower and upper-secondary levels [33]. 

#### 2.2.3. Recruitment

Schools were recruited by contacting principals and sending them an information letter about the study and its aims, which was followed by a meeting with the researcher. After a decision that a school would participate, the principals then informed the teachers in the two web program schools about the study, and each teacher could decide if they wanted to collaborate, cooperate and be involved in the web program as a tutor or not.

Adolescent recruitment at the three schools occurred during the researcher’s visit in mid-May 2013 (see also as a baseline). It included an oral presentation of the study. Adolescents and their guardians received written information about the study (the overall aim, study sites, target group, procedure, funding, collaborators, approving body, volunteer participation, anonymity, contact details of the persons in charge). Informed consent forms were delivered to the adolescents, and each adolescent who was willing to participate signed a written consent form and filled in the survey questionnaires. 

Teacher recruitment was organized at the same time at the two program use schools for teachers who were invited to act as program tutors for the adolescents. The study aim and procedures were described to them, and written information leaflets were delivered as well as consent forms and survey questionnaires.

### 2.3. Web Program

DepisNet-Thai is an expansion and implementation of good practices obtained from previous web programs for persons with mental health problems (ISRCTN74919979, ISRCTN80379583) [34,35]. Content of DepisNet-Thai was developed based on discussions with adolescents in Thailand showing stress mainly to be a problem. Adolescents’ high stress levels are associated with having a high number of depressive symptoms [36]. The aim of DepisNet-Thai is to support adolescent wellbeing in Thailand using alternative ways of delivering the program in schools and interacting with adolescents. It was either used in small groups (intervention group) or independently (active control group). The third group was passive control. The outcomes for each group were measured at the baseline and at the follow-up, at the beginning of August 2013 (11 weeks from the baseline assessment).

The content and theoretical background of the program is based on Garcia’s [37] construction of adolescent coping. The literature review shows that a wide range of stress-related risks or conditions in adolescence are developmental stressors (e.g., peer relationships, school accomplishments, physical and emotional changes), or environmental and contextual stressors (e.g., family separation, discrimination). How adolescents cope or respond to these stressors may influence their wellbeing [37]. In addition, emotion-focused coping involves responses such as expressing one’s emotions, seeking solace and support from others, and trying to avoid sources of stress [38]. In dealing with these stressors, adolescents’ self-management and coping capabilities often depend on their intrinsic motivation, which can be supported by increasing their positive feelings of autonomy, competence, and being connected [39].

DepisNet-Thai is a web program with individual user accounts and access codes, which were, in this case, delivered to participants by email. Usernames, rather than real names, were used in the learning management system, Moodle, which was administered by the University of Turku. In addition to a two-week orientation, DepisNet-Thai includes five weekly modules (Psychological stress, Physical wellbeing, Me and my family, Me and my friendships, Me and my society). Each module lasts about 50 min, and the total program takes seven weeks to complete if a two-week orientation is included. Adolescents had weekly exercises accessible on their own and at any time, that aimed to support their self-reflection skills and provide self-management skills.

A handbook for DepisNet-Thai was developed for tutors in the Thai language for both schools (intervention group, active control group). Teachers were trained to run the program and how to act as a tutor. They were also instructed to guide adolescents in answering contributory questions, read the students’ exercises, interact in discussions, and provide positive and supportive feedback of the exercises before the next module began. Moreover, tutors were instructed to monitor adolescents, checking that they had completed and returned their exercises according to the instructions. They were also guided in how to provide encouraging support that increases adolescents’ motivation to complete their exercises.

#### 2.3.1. Intervention Group

Adolescents used the DepisNet-Thai in small groups where participants were able to peer-network online with each other. The intervention group included 4 small groups, 11–14 adolescents per group and one teacher/tutor per group. During the first session, adolescents were asked to identify their problems or concerns related to stressing situations and find information and responses to their concerns via links to reliable Thai web sites, Thai or English literature and PowerPoints. Exercises were delivered in groups and the group members interacted with each other. Detailed description of the program and exercises are presented in Table S1: Description of a web program and Table S2: Description of group exercises.

#### 2.3.2. Active Control Group

Adolescents used DepisNet-Thai independently; they worked with the exercises alone and returned them in Moodle. One teacher/tutor supported and oversaw 14 adolescents in their giving of feedback and completing of exercises.

#### 2.3.3. Passive Control Group

Adolescents did not use or have access to the DepisNet-Thai.

### 2.4. Outcomes 

#### 2.4.1. Depression

The Patient Health Questionnaire (PHQ-9) measures signs of depression among adolescents [40]. It includes items that ask respondents to indicate on a four-point scale how often they have been bothered by any of the problems in the previous two weeks (0 = not at all; 3 = nearly every day). The higher the score, the more severe the depression symptoms are (range 0–27). Moreover, a tenth item asks respondents, if they indicated that they had experienced any of the problems in items 1–9, how difficult these problems have made it for them to manage their responsibilities (0 = not difficult at all, 3 = extremely difficult). According to Kroenke et al. [41], the instrument has been evaluated to be reliable. PHQ scores ≥ 10 have a sensitivity of 88% and a specificity of 88% for major depression. Regarding the Thai version of the instrument [42], internal consistency is satisfactory (Cronbach’s alpha = 0.79) and specificity is very high (0.98). The Thai version was used in our study with the permission of the copyright holder. 

#### 2.4.2. Stress

The Perceived Stress Scale (PSS) by Cohen et al. [43] assesses the degree to which people perceive their lives as stressful; unpredictable, uncontrollable, and overloaded in the previous month. A five-point scale for the individual items was used (0 = never, 4 = very often). The scale included four reversed items. The total score ranges between 0 and 40; the higher the score, the more the respondents perceive their lives to be stressful. The Cronbach’s alpha of the PSS-10 has been evaluated to be >0.70, and the test-retest reliability meets the criterion of >0.70 [44]. The Thai version T-PSS-10 by Wongpakaran and Wongpakaran [45] was used with the permission of the Thai authors. It has been demonstrated to be a reliable and valid instrument within the Thai culture.

#### 2.4.3. Satisfaction

The Client Satisfaction Questionnaire (CSQ-8) [46] used in this study measured satisfaction with services provided for adolescents. The instrument consists of items asking clients to rate the services on a four-point Likert scale (1 = quite dissatisfied, 4 = very satisfied). An overall score is produced by summing all item responses. Scores range from 8 to 32; the higher the score, the more the clients indicate satisfaction. In a variety of studies, the internal consistency of the instrument, as measured by a coefficient alpha, has ranged from 0.83 to 0.93. The eight items in the instrument are based on their factor loadings [47]. The CSQ-8 was used with the permission of the copyright holder. 

#### 2.4.4. Acceptance

Acceptance of DepisNet-Thai was assessed based on logged activity of the two web program groups. This activity includes the total number of adolescents and teachers logging onto the program during the study period, the number of activity logs in the program, and the number of activity logs in each module.

#### 2.4.5. Usability Feedback

Usability feedback of DepisNet-Thai was queried after eight weeks of use by the two web program groups. A survey included five questions, according to the Technology Acceptance Model [48], which has been used to describe the adoption of technology [49] and explain users’ behavior in IT implementation [50]. The questionnaire included dichotomous (yes, no) questions asking users the following questions: (1) were they satisfied with the program, (2) was it useful, (3) was it easy to use, (4) did it cause any harm, and (5) would they use the program in the future. Further, there were open-ended questions in which participants were able to provide more feedback on the usability. 

### 2.5. Sample Size 

A formal power calculation was not conducted because of the feasibility study [51]. According to a previous study [52], given the effect size of 0.64, with a statistical power of 0.8 and a confident level of 0.05, a sample of about 138 subjects is needed. Our sample size is quite similar to those of previous studies in this area [52,53,54,55,56] and is usable with this study design. 

### 2.6. Randomization

Individual randomization for adolescents was not conducted because it was not feasible given the possible information flow between the adolescents [57]. Therefore, the trial was undertaken in three conveniently sampled high schools. The schools were assigned into one of three groups based on each school’s own decision: (1) a school where adolescents (N = 60) used the web program in small groups (intervention); (2) a school where adolescents (N = 60) used it independently (active control); and (3) a school in which adolescents (N = 60) did not use the program at all (passive control). The allocation of the schools was not concealed, and researchers, adolescents, the teachers/tutors and a statistician were aware which groups were in which schools. 

### 2.7. Statistical Methods

Descriptive statistics (frequencies, percentages, means, standard deviations) were calculated for adolescents and teachers’ demographic information such as age, gender, adolescents’ regular school attendance (yes, no), previous use of mental health services (yes, no), and teachers’ working experience in years. For the PHQ-9 scale, a total score was formed by summing the values of all items. For the PSS-10 scale, four negative scores were recoded, and the total score was then formed by calculating a value for each of the 10 questions. For the CSQ-8 scale, an overall score was produced by summing all item responses.

Skewness was checked to be in the same direction in groups that were compared. As parametric tests have more power to reveal statistically significant differences or associations between groups, and should be used when possible, two measurements were first compared with paired T-tests. To evaluate the effect of background variables over time, a repeated measures Analysis of Variance (ANOVA) with one background variable in each model was used. Fisher’s exact tests were used to find out the dependence between categorical background variables and PHQ-groups. Differences in background variables between schools were tested using Chi Square tests and a one-way ANOVA.

A sensitivity analysis (repeated measure analysis of variance) was performed comparing the users and non-users (e.g., adolescents who did not log on to the web program at all in either of the two groups). The users of the web program were therefore collapsed into one group regardless of their initial group allocation for the purpose of examining program impact. F-tests were used to measure differences between the groups.

The data regarding acceptance of the program was analyzed with descriptive statistics (f, %) for the number of adolescents and teachers using the program in the two web program groups, the frequency of use, and the favorability of the topics. Possible differences between logging on of adolescents between the groups were compared with Chi Squares tests. Usability feedback was analyzed with questions posed to the adolescents and teachers.

Data analyses were performed using the statistical program SPSS 22.0 (SPSS Inc., Chicago, IL, USA). Observed significance levels <0.05 were considered to be statistically significant. Exact *p*-values are reported.

### 2.8. Ethical Issues

Local authorities from the Prince of Songkla University in Thailand were consulted about the study proposal and its attachments. The principals of the participating schools guaranteed permission for the study to be conducted. Ethical approval was granted from the Ethics Committee of the University of Turku (17/2016), and the entire procedure has conformed to the standards currently applied in the study’s country of origin. 

Ethical guidelines and the basic principles of research ethics [58] were followed throughout the study. Any regulations addressing the conduct of a vulnerable population such as minors were taken into account. Participants decided for themselves whether to participate or not. It was assured that participation was voluntary and refusal to participate in the study would not in any way affect adolescents in their studies or teachers in their work. 

Teachers/tutors were also instructed to pay special attention to the adolescent’s writings, including any indication of suicidal thoughts or self-harm (serious adverse events). Anonymity and confidentiality issues were emphasized throughout the study, as usernames were used rather than real names. The learning management system, Moodle, is Secure Sockets Layer (SSL)-protected with usernames and passwords. The data were transferred, analyzed and reported in a way that anonymity could be preserved. Data were kept in a locked and safe place; only the researchers and the statistician were able to access the data. 

## 3. Results

### 3.1. Participant Flow

**Figure 1:**
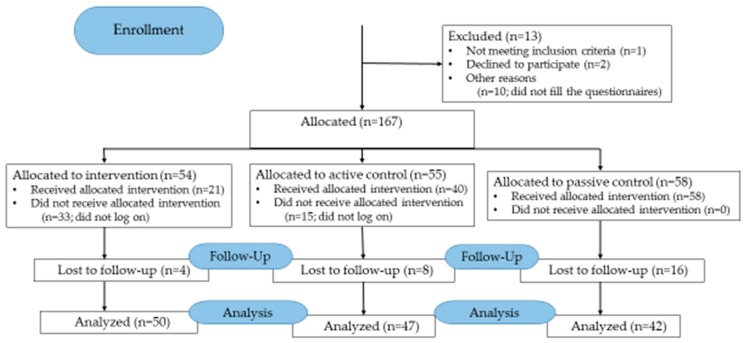
Flow diagram of the adolescents (CONSORT 2010 Flow Diagram).

A total of 180 adolescents were potentially eligible for the study, and 167 (93%) of them were non-randomly assigned to the groups: intervention (n = 54), active control (n = 55), and passive control (n = 58). Thirteen were ineligible (Figure 1). 

### 3.2. Baseline Data 

A description of the adolescents and sample characteristics for each group at baseline are provided in Table 1. Adolescents were around 16 years old, more than half of them were females, almost all of them had regular school attendance and hardly anyone had used mental health services before. In general, they had minimal depression symptoms, their stress scores were about 16 (out of 40) and their satisfaction scores ranged between 18 and 24 (out of 32). There were statistically significant differences between the intervention, active control and passive control groups in age (15.76 vs. 15.78 vs. 16.22, *p* = 0.001), % of females (64.2% vs. 54.5% vs. 86.2%, *p* = 0.001) and satisfaction scores (18.72 vs. 17.53 vs. 23.81, *p* = 0.001). The passive control group contained a larger portion of girls, and the adolescents in that group were older and more satisfied with health and human services compared to the adolescents in the intervention and active control groups. 

Out of 12 teachers, 10 (83%) participated in the study; six were at the school with the intervention and four were at the school with the active control group. The teachers at school offering the intervention were all females, they were older, and they had more teaching experience compared to those at the school with the active control group. Teachers were also queried if there are methods to support adolescent wellbeing and prevent mental health problems at school. The characteristics and answers of the participating teachers (n = 10) are summarized in Table 2. 

### 3.3. Numbers Analyzed

All adolescents assigned to a condition were included, regardless of their degree of participation. The follow-up was conducted with the remaining adolescents in the intervention (50/54, 93%), active control (47/55, 85%) and passive control (42/58, 72%) groups whether they received the allocated program or not. The number of adolescents included at baseline and in the follow-up analysis of depression, stress and satisfaction were n = 47, n = 50 and n = 26 in the intervention group, n = 46, n = 47 and n = 15 in the active control group, and n = 42, n = 42 and n = 16 in the passive control group. Missing data were not imputed. Moreover, web program users were compared to non-users.

### 3.4. Outcomes 

#### 3.4.1. Depression 

Mean changes in depression scores were 0.3 in the intervention group, 0.9 in the active control group, and 0.8 in the passive control group between the baseline and follow-up measurements. The changes from baseline to follow-up did not differ significantly between the groups (*p* = 0.594) (Table 3).

#### 3.4.2. Stress

Mean changes in stress scores between baseline and follow-up were as follows in the intervention, active control and passive control groups: −0.7 vs. 0.4 vs. 0.9. The changes between baseline and follow-up did not differ significantly between the groups (*p* = 0.178). 

#### 3.4.3. Satisfaction

Mean changes in scores of satisfaction with provided services between baseline and follow-up were as follows in the intervention, active control and passive control groups: −0.1 vs. −0.6 vs. 3.8. Changes in satisfaction scores between baseline and follow-up did not differ significantly between the groups (*p* = 0.101).

#### 3.4.4. Comparison between Program Users and Non-Users

Web program users (n = 61, mean age 16.02, females 62.3%) were compared to non-users (n = 48, mean age 15.46, females 54.2%). The 48 adolescents who did not use the program consisted of 33 adolescents in the intervention group and 15 adolescents in the active control group. The adolescents who used the program had lower depression (6.8 vs. 7.8, *p* = 0.194) and stress scores (15.4 vs.16.5, *p* = 0.322), and they were more satisfied with the services (20.4 vs. 20.2, *p* = 0.785) compared to non-users at the follow-up, even though differences between the two groups did not differ significantly.

#### 3.4.5. Acceptance of the Program by Adolescents 

Out of the 120 adolescents invited to use the program at the two schools, 109 (91%) participated in the study. Further, 61 out of 109 (56%) logged on to the program, but activity differed between the groups (n = 21, 39% in intervention; n = 40, 73% in active control, *p* = 0.001). 

Notably, the number of adolescents who logged on to the program decreased over the time in both groups. The frequency of users was highest for the first module and lowest for the last module (Figure S1: Number of adolescents logged on in weekly modules). The analysis of activity logs in each module showed that intervention group activity was the highest in the first module and lowest in the fourth module. In the active control group, adolescent activity was the highest in the fourth module. (Figure S2: Number of activity logs throughout the program.) Adolescents in the intervention logged less to the program in all the modules compared to adolescents in the active control. 

The analysis of activity logs revealed that the range of total activity logs was quite similar between the intervention (range 2–68) and active control (2–64) groups. When the total number of activity logs were compared, adolescents in the intervention group were found to log on less often than those in the active control group (578 vs. 936, *p* = 0.003). In addition, each adolescent in the intervention group visited fewer modules ((zero modules n = 33 vs. n = 15), (one module n = 9 vs. n = 8), (two modules n = 6 vs. n = 9), (three modules n = 3 vs. n = 6), (four modules n = 2 vs. n = 3), (five modules n = 0 vs. n = 11), (six modules n = 1 vs. n = 3)) than those in the active control group (totally 44 vs. 129, *p* < 0.001).

#### 3.4.6. Acceptance of the Program by Teachers 

Out of the 10 participating teachers, three of them logged on to the program (30%), and they were all involved with the active control group. None of the six teachers in the intervention group logged on to the program. The analysis of activity logs revealed that the total numbers of activity logs for the active control group teachers were 1, 107 and 141. The analysis of activity logs in each module showed that the number of teacher logins in the active control group was altogether 249 visits, which included six visits to the orientation, 38 visits to Module 1 (Psychological Stress), 77 visits to Module 2 (Physical wellbeing), 127 visits to Module 3 (Me and my family), one visit to Module 4 (Me and my friends) and zero visits to Module 5 (Me and my society). 

#### 3.4.7. Usability Feedback 

Adolescents provided feedback about the program (49 in the intervention group, 47 in the active control group). In general, three-fourths of all web program users (77%) were satisfied with DepisNet-Thai regardless of their group. Less than one-third (27%) found the program useful, and 67% thought that the program was easy to use. Four adolescents (4%) expressed that the program was harmful. Over half (57%) stated that they would use the program in the future. No statistically significant differences were found (see Table 4) when comparing feedback of the adolescents in the intervention group and that of those in the active control group.

Of the 10 teachers, all were satisfied with DepisNet-Thai. Four found the program to be useful, half of them evaluated it as easy to use and two considered it to be harmful in some way. All of them would use the program in the future. 

## 4. Discussion

The aim of this quasi-experimental feasibility study was to study the impact, acceptance and usability of a web program, and make a comparison between program users and non-users. We did not find any statistically significant changes between the groups regarding depression, stress or satisfaction. There have been discrepancies in the results of previous studies regarding changes in adolescents’ stress scores. In a systematic review and meta-analysis of web programs to support adolescent depression [59], a statistically significant improvement was found (10 studies) in the intervention group at the end of the intervention and 6 months after the intervention. However, there were no differences (8 studies) in users’ stress scores [59]. One reason for users to rate the web-based program’s effectiveness as being low may be because of adolescents not completing the program [60] which is understandable because of use being voluntary. As an outcome of incompletion, a user would receive an insufficient dose of the program [18]. We also found that the adolescents who logged on to the program and used it were older and benefitted from it in terms of decreased depression and stress scores and increased satisfaction, although the differences between the users and non-users were not significant. Previous studies have also shown that adolescents who complete the program [19,61] and use the program more frequently [19] derive the greatest benefit from it. Therefore, acceptance of the program needs additional focus. 

In our study, we found that acceptance was lower among adolescents who were in small groups compared to those who participated in the program independently. The result is in line with a study by Barak et al. [10], in which individually used online programs were more effective than group programs. In our study, the frequency of logging on among adolescents decreased over time in our study period, and was highest in the first module and lowest in the last module. A decrease in use of the web programs over time has been found previously in studies where non-adherence and high dropout rates have been significant hurdles [12]. Around 70% of participants using a self-directed IT program for depression in school have completed less than three of the modules [62], less than 10% logged on and only few proceeded beyond the first module [18]. The low adherence rate in our study is of interest since the program content was developed based on adolescent interviews about their current needs, and was theoretically based on factors related to social stressors, relevant to adolescent coping [37]. 

Further, adolescents’ log activity decreased more among adolescents who used the program in small groups. During the development process of the program, adolescents suggested the importance of discussion forums for exchanging information and ideas between students and teachers. However, the adolescents in the current study did not use the program in small groups. The result is of interest since Goldbaum et al. [63] reported that students seek support and protection and discuss everyday events, personal concerns and problems with their peers. Adolescents are also willing to build up their social relationships [64]. However, we can ask whether a forum for openly discussing and sharing inner thoughts with peers is too intimate of a way to seek help for daily issues. If a teacher is also a tutor, it may feel uncomfortable for an adolescent to share personal issues, even if identities are kept anonymous, keeping in mind that that teacher would also be responsible for the adolescent’s grades in school. Another reason for the adolescent non-adherence to the program may be that none of the teachers in the small group logged on to the program and supported the adolescents even if they were required to. In a previous study, the completion rate was also lower without the support [14].

In total, fewer than one-third (27%) of the adolescents found the program useful although 67% thought that it was easy to use. The results are of interest since the program was developed to respond to the requirements of Thai adolescents by defining the needs and solutions for new programs. Information about adolescent studies and education, tuition, study guidance and schooling were highlighted as important by adolescents during the development process. Adolescents desire accessible and usable information that they find important and advantageous [65]. In our study, adolescents also desired trustworthy information and wanted to know official sources of the information. On the other hand, if adolescents were not depressed or stressed, it is possible that the low adherence may be because of that and have impact on their feedback of the program usefulness. Our feedback questionnaire may have caused confusion in what kind of feedback to provide if adolescents or teachers had not used the program—whether to provide feedback based on actual use or in theory.

Our study also yielded some limitations. First, a significant limitation is that the participants were non-randomly assigned to the groups which would have prevented the selection bias [66]. Second, not all the program users’ emails worked, so adolescents and teachers had to be contacted several times at the beginning of the program, and this may have reduced acceptance of the program. Third, there were semester examinations going on at the schools, which may have drawn adolescents’ interest away from the program. Fourth, some of the program exercises were returned in an unreadable format, and adolescents did not return them again in a readable format, even if asked to, which could have insured the accidental bias [66]. Fifth, some of the returned exercises in the intervention group were deleted by mistake in the first week of the program when transforming the exercises from one format to another. Adolescents did not return their exercises again despite several pleas for them to do so. This could have hindered their trust in the program and reduced their motivation to use it. Some of these practical difficulties reveal that IT programs may work only when they have been assimilated into practice and delivered through the coordinated actions of the personnel [67]. The program was also planned in a way that the adolescents were able to use it at school (50 min per module) and get credits of participation, but it is not clear how it was carried out in daily school life which may have reduced acceptance. The length of the program was also modified after recruitment and baseline measurement. Orientation took two weeks and because of email problems and semester examinations, the length of the first module took three weeks instead of one. Moreover, data was biased as passive control adolescents had statistically significant differences in some of the factors at baseline compared to adolescents in other groups. It might be because the passive control group contained a larger portion of girls and the adolescents in that group were older compared to the adolescents in the other two groups. Therefore, differences between schools may have affected results even though we aimed to obtain homogenous data (purposive convenience sampling) regarding school size, type, location, curricula, and teachers’ intention to collaborate, cooperate and be involved in the web program as a tutor. However, randomizing at the school level could have at least reduced the selection bias [66].

Still, mental health promotion using a variety of programs is an investment in the future. It is important that mental health promotion is being done in a place where adolescents spend their time [68,69]. According to Lillevoll et al. [18], providing mental health programs within the school environment is likely to ensure better uptake, especially among senior high school students. Interest towards a program is a good sign as adolescents may have difficulties in recognizing symptoms or they may have a preference for self-reliance [70]. This study has an important role in Thailand where only a small amount of research has been conducted about the potential use of modern methods to support adolescents’ mental health and general wellbeing in school environments. Preventive programs and practices should therefore be developed and implemented [71], especially in places such as schools.

## 5. Conclusions

This study provides information worthy of future research about the specific tool used at schools in Thailand. It also yields more knowledge for teachers, organizations, society and policymakers of IT adoption, its use in school environments and information about implementation in real practice. It is important to evaluate the procedures of a program in order to assess its impact.

Although we could not prove that the DepisNet-Thai program is beneficial in terms of decreased depression and stress scores and increased satisfaction among users in comparison to those who did not log on, we identified that independent use of the program was more popular than use in small groups. Our study indicated that health-related programs and applications need careful training and available local support when introduced into practice in order to be potentially used. We still believe that IT-based programs have potential in school environments. However, they should be incorporated early at the elementary level [1]. More information is urgently needed about the factors that affect the use of such programs.

Indeed, one challenge is how to implement IT services into daily practices [72]. Administrative support from school leaders can therefore be pivotal to effective program implementation by identifying suitable personnel, allocating resources, and motivating and obtaining the commitment of the personnel [73]. It is of importance to the professionals working with adolescents to understand that support should be provided to adolescents in many ways, since the adolescents that use the program may gain benefits from it. Recent literature clearly indicates that mobile technology can be usable in low-income and middle-income countries if it is ensured that target users receive, engage with, and benefit from the technology interventions as intended [74].

## Figures and Tables

**Table 1:** Baseline characteristics of adolescents and assessment of differences between the groups.

Characteristics	Intervention n = 54				Active Control n = 55				Passive Control n = 58				*p*-Value
	n	%	Mean	SD	n	%	Mean	SD	n	%	Mean	SD	
Age			15.76	0.70			15.78	0.88			16.22	0.56	0.001
Female gender	34	64.2			30	54.5			50	86.2			0.001
Regular school attendance	53	100.0			54	98.2			56	96.6			0.395
Previous mental health service use	2	3.8			1	1.9			2	3.4			0.823
Depression score (PHQ-9)			8.07	4.61			7.62	3.05			7.79	4.08	0.833
Stress score (T-PSS-10)			15.87	4.91			15.45	4.37			16.55	5.47	0.493
Satisfaction score (CSQ-8)	29	53.7	18.72	6.09	17	30.9	17.53	7.76	32	55.2	23.81	2.60	0.001

SD: Standard Deviation; PHQ-9: The Patient Health Questionnaire; T-PSS-10: The Perceived Stress Scale; CSQ-8: The Client Satisfaction Questionnaire.

**Table 2:** Teachers’ characteristics at baseline.

Characteristics	Baseline	
	Intervention %	Active Control %
Age	n = 6	n = 4
Under 30	50	75
30–39 years	0	25
40–49 years	17	0
Over 50	33	0
Gender	n = 6	n = 4
Male	0	25
Female	100	75
Teacher working experience	n = 3	n = 3
Under 10 years	0	100
10–19 years	0	0
20–29 years	33	0
over 30 years	67	0
Does the school have methods to support adolescent wellbeing and prevent mental health problems	n = 5	n = 4
Yes	0	75
No	100	25
Are teachers satisfied with methods	n = 3	n = 4
Yes	67	75
No	33	25
Does the teacher have useful experiences of methods	n = 3	n = 4
Yes	0	75
No	100	25
Are methods easy to use	n = 3	n = 4
Yes	67	75
No	33	25
Are methods harmful	n = 3	n = 4
Yes	0	50
No	100	50
Does the teacher have intentions to use the methods in the future	n = 3	n = 4
Yes	67	100
No	33	0

**Table 3:** Adolescents’ depression, stress and satisfaction scores.

	Intervention (54/50)			Active Control (55/47)			Passive Control (58/42)			
Outcome	BL Mean (SD)	FU Mean (SD)	Change Mean	BL Mean (SD)	FU Mean (SD)	Change Mean	BL Mean (SD)	FU Mean (SD)	Change Mean	*p*-Value
**PHQ-9** depression	n = 54 7.8 (4.5)	n = 47 7.5 (3.7)	0.3	n = 55 7.8 (3.0)	n = 46 6.9 (3.5)	0.9	n = 58 7.8 (3.7)	n = 42 7.0 (3.1)	0.8	0.594
**T-PSS-10** stress	n = 55 15.6 (4.9)	n = 50 16.3 (5.1)	−0.7	n = 55 15.9 (4.3)	n = 47 15.5 (4.8)	0.4	n = 58 16.1 (4.9)	n = 42 15.2 (5.0)	0.9	0.178
**CSQ-8** satisfaction	n = 29 19.7 (5.6)	n = 26 19.8 (3.4)	−0.1	n = 17 18.8 (7.4)	n = 15 19.4 (3.5)	−0.6	n = 32 23.4 (2.5)	n = 16 19.6 (5.2)	3.8	0.101

BL = Baseline; FU = Follow-up; SD: Standard Deviation; PHQ-9: The Patient Health Questionnaire; T-PSS-10: The Perceived Stress Scale; CSQ-8: The Client Satisfaction Questionnaire.

**Table 4:** Adolescents’ feedback of the program.

Statement		Intervention n (%) *		Active control n (%) *		
	N		n		n	*p*
Have you been satisfied with the program?	96	34 (69)	49	40 (85)	47	0.067
Have you experienced the program to be useful?	96	16 (33)	49	10 (21)	47	0.210
Is the program easy to use?	96	30 (61)	49	34 (72)	47	0.248
Has the program caused any harm to you?	96	1 (2)	49	3 (6)	47	0.287
Would you use this kind of program in the future?	95	27 (56)	48	27 (57)	47	0.906

* responses n (%) represent answer ‘yes’.

## Supplementary Materials

The following are available online at https://www.mdpi.com/1660-4601/16/14/2473/s1, Figure S1: Number of adolescents logged on in weekly modules, Figure S2: Number of activity logs throughout the program, Table S1: Description of a web program, Table S2: Description of group exercises.
