# Supplementary material for: Impact of a Web Program to Support the Mental Wellbeing of High School Students: A Quasi Experimental Feasibility Study
Source: Int J Environ Res Public Health. 2019 Jul 11;16(14):2473. doi: 10.3390/ijerph16142473 (PMC6679030; doi:10.3390/ijerph16142473)
Supplement: Supplementary file 1 [file ijerph-16-02473-s001.pdf]

Supplementary Materials

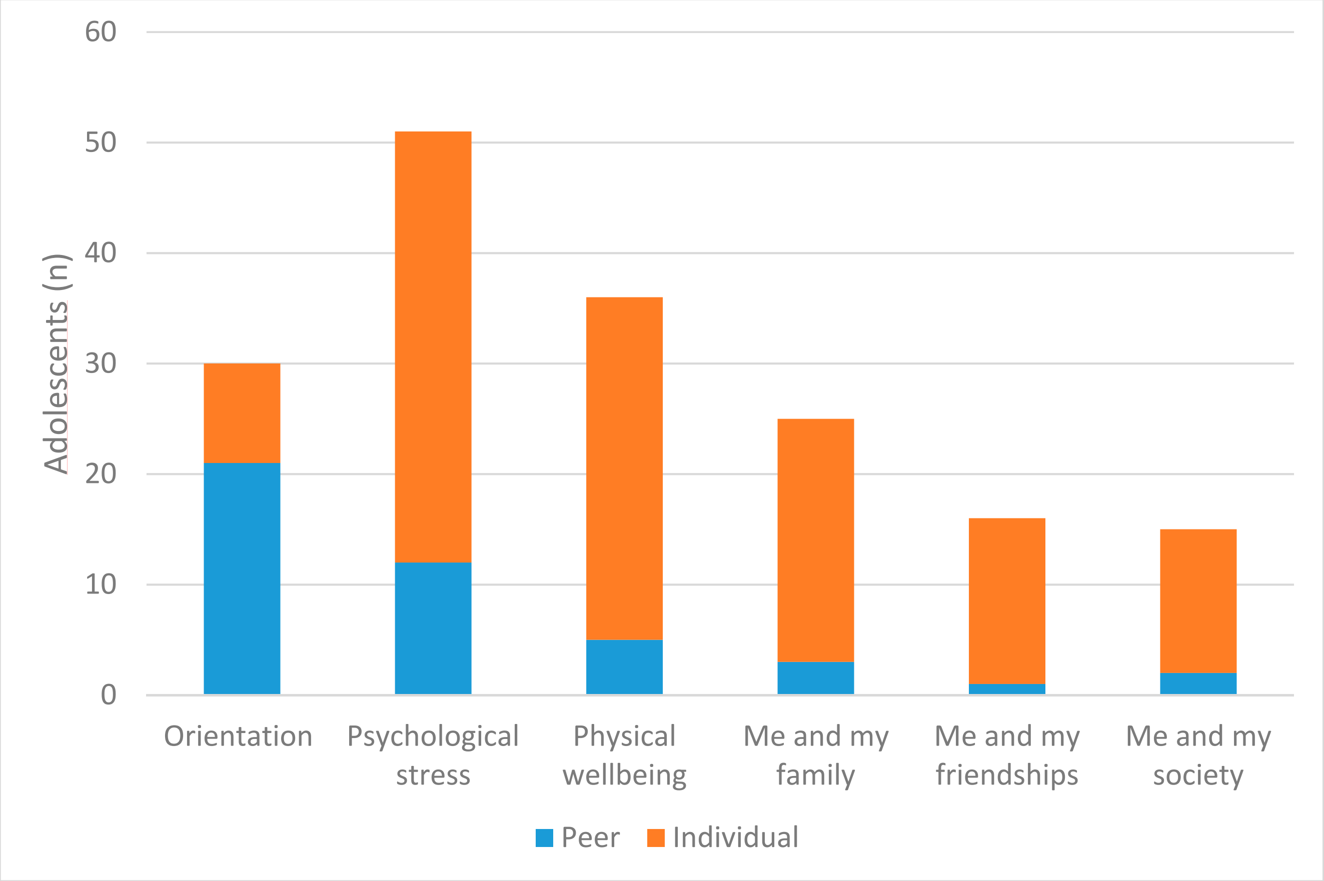

Figure S1. Number of adolescents logged on in weekly modules.

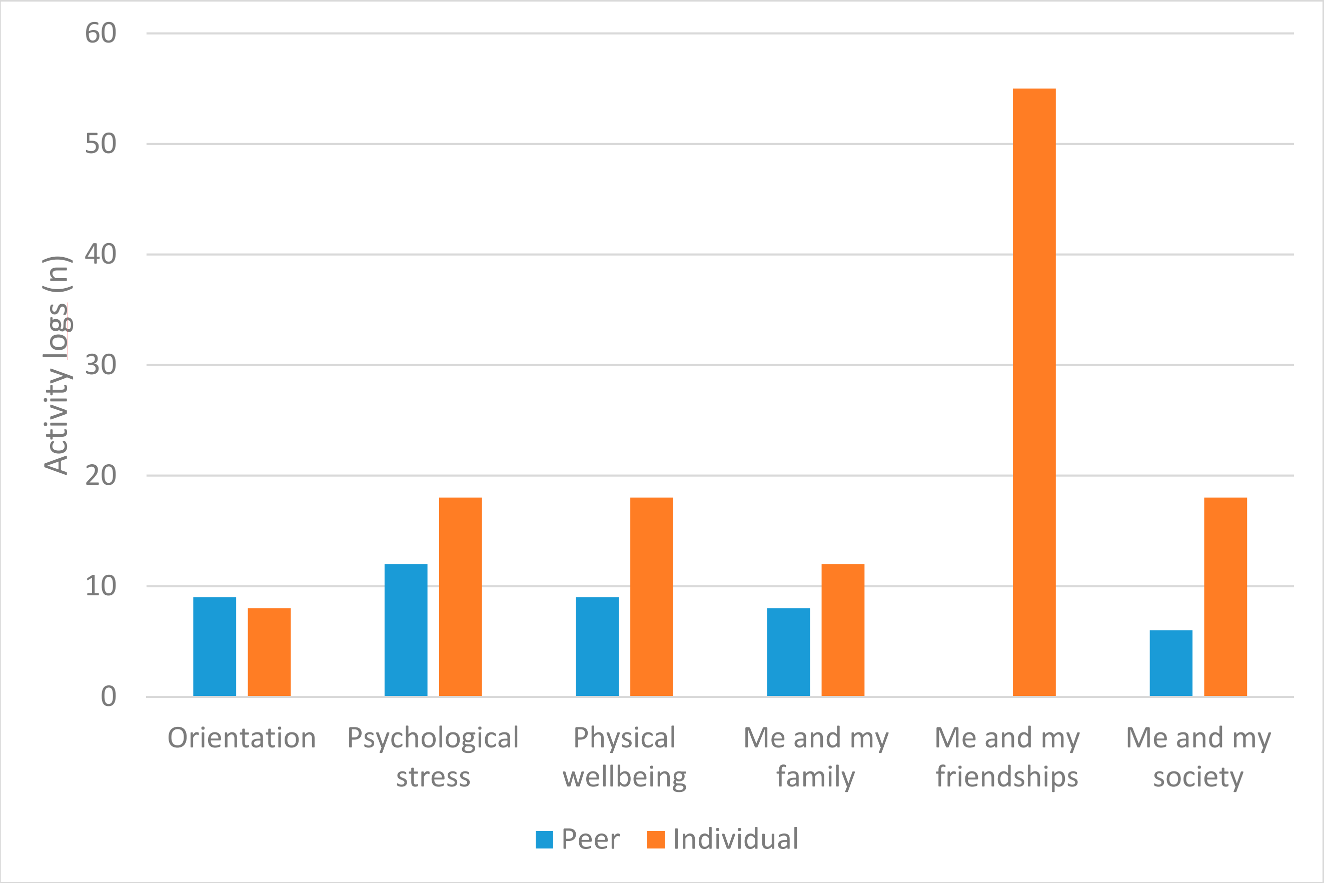

Figure S2. Number of activity logs throughout the program.

Table S1. Description of a web program.

Module	Content	Adolescent Exercise
Orientation	• Welcoming words<br>• Security, net etiquette and copyright<br>• Description of the program<br>• News<br>• Discussion forum<br>• Introduction of program leaders and tutors<br>• Information about Moodle	
Psychological stress	• Information about why the topic is important<br>• Ways to identify stressful situations<br>• Additional information about stress<br>• Prevention of mental health problems	A short reflection of no more than 500 words about stressful situations and a strategy plan for the future.
Physical wellbeing	• Information about what it means to become an adult<br>• Additional information about appearance and health<br>• Information about hobbies and exercises<br>• Information about sexuality	Writing in a "diary" with paper and pencil about any worries or concerns from the previous week, possibly connecting them with the program information given that week, and writing of a plan to reduce physical stressors.
Me and my family	• Information about adolescents as family members	Reading an article about how parents are guided to cooperate with their children, discussing

	• Information about how to communicate in a positive way	with other adolescents in the discussion forum about points of disagreement or content that should be added.
Me and my friendships	• Information about interpersonal relationships<br>• Information about problems with relationships<br>• Information about bullying, cyberbullying and social media and how to cope<br>• Ways to prevent bullying and cyberbullying	Offering an illustration (short text, pictures, lyrics, poems, figures, Youtube videos, etc.) to describe concrete and practical tips on how to prevent bullying and cyberbullying in real life and in social media.
Me and my society	• Information about the role of adolescents in society<br>• Choosing a career and coping with this process	Providing feedback about the program.

Table S2. Description of group exercises.

Module	Intervention Group	Active control Group
First module	Adolescents added their stress exercise to a discussion forum and shared it with the others. Group members provided comments to each other's writings and supported each other.	Adolescents provided an exercise about stress.
Second module	Adolescents shared their diary exercises and thoughts (last week's worries and concerns, plans to reduce physical stressors) with the others in the discussion forum. They also commented on each other's plans and provided supportive feedback.	Adolescents wrote a plan for themselves.
Third module	Adolescents discussed in the forum about the exercise (article about how parents are guided to cooperate with their children) and returned it in the program's wiki, and together developed a guide for parents on how to cooperate with their children.	Adolescents discussed with each other in the discussion forum about the guide to be read. Moreover, they commented in Moodle on how parents should cooperate with their children.
Fourth module	Adolescents discussed their tips for how to prevent bullying and cyberbullying, offered a short illustration about it, and read and comment on each other's tips with positive feedback.	Adolescents used the forum to discuss their tips on how to prevent bullying and cyberbullying and also returned a short exercise about it.
Fifth module	Adolescents provided feedback on DepisNet-Thai and shared it with the others.	Adolescents provided feedback on DepisNet-Thai.
